# Oral and vaginal microbiota in selected field mice of the genus *Apodemus*: a wild population study

**DOI:** 10.1038/s41598-020-70249-x

**Published:** 2020-08-06

**Authors:** Tereza Matějková, Petra Hájková, Romana Stopková, Michal Stanko, Jean-François Martin, Jakub Kreisinger, Pavel Stopka

**Affiliations:** 1 Department of Zoology, Faculty of Science, Charles University, BIOCEV, Vestec Czech Republic; 2 Institute of Vertebrate Biology, Czech Academy of Sciences, Brno, Czech Republic; 3 Institute of Parasitology, Slovak Academy of Sciences, Košice, Slovakia; 4 Montpellier-SupAgro, UMR Centre de Biologie Pour La Gestion Des Populations, Montferrier-sur-Lez, France

**Keywords:** Microbiome, Microbial ecology

## Abstract

Animal-associated microbiota is expected to impose crucial effects on the host’s fitness-related performance, including reproduction. Most research to date has focused on interactions between the host with its gut microbiota; however, there remain considerable gaps in knowledge regarding microbial consortia in other organs, including interspecific divergence, temporal stability, variation drivers, and their effects on the host. To fill these gaps, we examined oral and vaginal microbiota composition in four free-living mouse species of the genus *Apodemus*, each varying in the degree of female promiscuity. To assess temporal stability and microbiota resistance to environmental change, we exposed one of the species, *Apodemus* *uralensis*, to standardized captive conditions and analyzed longitudinal changes in its microbiota structure. Our results revealed the existence of a “core” oral microbiota that was not only shared among all four species but also persisted almost unchanged in captivity. On the other hand, vaginal microbiota appears to be more plastic in captive conditions and less species-specific in comparison with oral microbiota. This study is amongst the first to describe oral microbiota dynamics. Furthermore, the vaginal microbiota results are especially surprising in light of the well-known role of stable vaginal microbiota as a defense against pathogens. The results indicate the existence of diverse mechanisms that shape each microbiota. On the other hand, our data provides somewhat ambiguous support for the systematic effect of phylogeny and social system on both oral and vaginal microbiota structures.

## Introduction

Symbiotic microbiota have adapted to the diverse conditions found within animal bodies and have a profound effect on many aspects of an individual’s life, including its health^1^ and fitness^2,3^. Species-specific microbiota alter the body’s odor^4^, protect its host against pathogens^5,6^, and influence mating preferences^7^ or behavior in general^8^. However, most research to date has focused on gut microbiota^7,9,10^; thus, most body parts remain unexplored in non-human hosts. For example, skin microbiota^6^ or vaginal microbiota^11^ also contribute to the host’s health and fitness and it has been shown that tissue-specific differences exist in the expression of antimicrobial proteins, which presumably regulate the abundance and diversity of microbiota along the eyes–nose–oral cavity–gut axis^12–14^. As such, the detection of particular factors that shape microbial composition in particular tissues is challenging.

Variation in microbiota between geographically distinct, but ecologically comparable, populations of the same species suggests that local environmental pools are important sources for host-associated microbiota^15^. Especially the diet seems to be crucial for the gut microbiota composition. The standardized diet in captivity not only alters the microbiota but also hinders the microbiota recovery after releasing back into the natural environment^16^. At the same time, however, environmental microbiota differ greatly from communities found in and on hosts bodies, which implies the existence of host-intrinsic filters that allow co-opting of narrow and highly-specific subsets of bacteria from the environmental pool^17^. Furthermore, some bacteria can be transferred through social contact between community members. In the case of stable social transfer, bacteria exhibit transgenerational inheritance, meaning that their phylogeny follows the biogeographic and phylogenetic history of their hosts^18^. Despite research efforts dedicated to uncovering the relative contribution of these factors to gut microbiota divergence at both the interspecific and interindividual levels^19^, how these factors shape host-associated microbiotas away from the digestive tract remains largely unknown.

Eurasian field mice (genus *Apodemus*) comprise four common species in Central Europe, *A. agrarius, A. flavicollis, A. sylvaticus*, and *A. uralensis*. These have adopted varying mating strategies ranging from monogamy to a high level of promiscuity^20^; interestingly, these strategies were then followed by further adaptations for a promiscuous or monogamous life. Monogamous *A. uralensis*, for example, show a longer-lasting sperm acrosomal reaction during fertilization, while reaction in the slightly more promiscuous *A. flavicollis* is a bit shorter, very fast in the even more promiscuous *A. sylvaticus*^21^, and extremely fast in the most promiscuous of all, *A. agrarius*^22^. These species also differ in social behavior, with promiscuous *A. sylvaticus* males investing less into the female during social allogrooming, with females rarely reciprocating^23^, while both males and females allogroom equally and reciprocally in the more monogamous *A. uralensis*^24^.

Different degrees of promiscuity and differences in mating and behavioral strategies make these four *Apodemus* species amenable for studying intra- and inter-specific differences in the structure of microbial populations. As oral contacts are frequently observed at both extremes (i.e., *A. sylvaticus* and *A. uralensis*) of these field mice^23,24^, we focused our investigations on oral and vaginal lavage samples, with the expectation that there would be detectable differences in microbiota composition. Subsequently, we assessed whether the community structure of oral and vaginal microbiotas were correlated and, using differential abundance analysis, we searched for species-specific bacteria within the vaginal and oral microbiota. Using time-series samples from *A. uralensis* transferred from the wild into captivity, we inferred temporal variation in the oral and vaginal microbiotas and their sensitivity to environmental change. Finally, we present a novel approach for delimitation of core microbiota based on a combination of data from free-living populations and a population exposed to experimental shifts in environmental conditions induced by captivity.

## Material and methods

### Animals and sample collection

A total of 96 adult *Apodemus* mice were sampled from sites in Slovakia [Šebastovce (48.654N, 21.272E), Kechnec (48.553N, 21.274E), Belža (48.586N, 21.271E), and Grajciar (48.596N, 21.265E)] in May 2014. In order to reduce the impact of geographical differences on microbiota, we studied populations of four *Apodemus* species known for syntopic occurrence in southeastern Slovakia. Therefore the selection of the sampling area was calculated. Of these, 28 individuals belonged to *A. agrarius* (15 females), 25 to *A. flavicollis* (9 females), 16 to *A. sylvaticus* (6 females), and 27 to *A. uralensis* (8 females). Within few hours after capture, animals were non-terminally sampled for oral and vaginal microbiome samples. Individual mouse was fixed by hand and oral cavity was flushed with 30–50 µl of sterile ddH_2_O and gently wiped with sterile nylon flocked swabs (FLOQSwab Minitip, Copan). Tip of the swab was immersed in PBS. Vaginal lavages were performed by gentle flushing with a pipette using 30 µl of sterile ddH_2_O. All samples were immediately frozen at − 20 °C. Sterile pipette tips with filters were used for all samples and the mice were handled using disposable laboratory gloves. All animals, except of 11 A. uralensis, were released back at the locality where they were trapped. The 11 *A. uralensis* were then transported to the animal facility at Charles University, Prague, and housed in small groups consisting usually of two males and two females in standard plastic cages (275 × 215 × 140 mm) with wire mesh lid and ad libitum access to water and food (ST1 food, Velaz, Prague, Czechia). There was no specific treatment neither for water nor food. Oral and vaginal samples were collected from the captive individuals in the same way as described above on the 42nd, 90th and 180th day of captivity.

### Microbiota profiling

The oral samples collected in ddH_2_O and PBS were merged for DNA extraction. Bacterial genomic DNA was extracted from the samples using the QIAamp DNA Mini Kit (Qiagen, Hilden, Germany), according to the manufacturer’s protocol. Based on our small pilot study where the mixed samples from several individuals were used, this kit was selected from a few other kits (e.g. QIAamp DNA Mini Kit, QIAamp DNA Stool Mini Kit, Powersoil DNA Isolation Kit from MOBIO) as the one with the highest amount of isolated DNA. The first step included lysis using 20 μl of lysozyme (140,000 U per sample; Elisabeth Pharmacon) for 60 min at 37 °C. The final elution of extracted DNA was performed with 80 μl of AE buffer. DNA concentration was measured using Qubit dsDNA HS Assay Kit. Primers covering the V3–V4 variable region on bacterial 16S rRNA (i.e., S-D-Bact-0341-b-S-17 (CCTACGGGNGGCWGCAG) and S-D-Bact-0785-a-A-21 (GACTACHVGGGTATCTAATCC)^25^) were used during the polymerase chain reaction (PCR) step. For demultiplexing, both forward and reverse primers were tagged by 10 bp barcodes. For the PCR (total volume 15 μl) , we used 7.5 μl of KAPA HIFI Hot Start Ready Mix (Kapa Biosystems, USA), 0.6 μM of each primer and 3 μl of DNA template in case of oral and 5 μl in vaginal samples (vaginal samples had lower DNA concentrations). PCR conditions were as follows: initial denaturation at 95 °C for 4 min followed by 35 cycles each of 98 °C (20 s), 61 °C (15 s), and 72 °C (40 s), and a final extension at 72 °C (5 min). Each sample was amplified in triplicate (technical replicates), and one replication from three was performed with different tag combination to check for consistency of sequencing. The PCR product, together with negative controls (PCR products for blank DNA isolates), were processed as described in^19^. Specifically, 1.5% agarose gel was used for the checking of products and the Qubit dsDNA BR Assay Kit was used for measurement of each PCR product's concentration. The samples were pooled at equimolar concentration and subsequently ran on 1.5% agarose gel. Bands of appropriate size were excised from the gel and following purification was done with the High Pure PCR product Purification Kit (Roche, Switzerland) according to the manufacturer’s instructions. TruSeq nano DNA library preparation kits (Illumina, USA) were used for a ligation of sequencing adaptors and sequencing of the resulting amplicon was performed on a single Miseq run (Illumina, USA) using v3 chemistry and 2 × 300 bp paired-end reads. We sequenced 181 samples of oral and vaginal microbiota, 133 of which were collected from wild populations of the four *Apodemus* species and 48 from *A. uralensis* following introduction to the breeding facility. The number of samples per species from the wild populations was as follows: *A. agrarius*—27 oral and 15 vaginal samples; *A. flavicollis*—25 oral and 9 vaginal samples; *A. sylvaticus* 16 oral and 6 vaginal samples; *A. uralensis* 27 oral and 8 vaginal samples.

### Bioinformatic analysis

Raw fastq files were demultiplexed and quality filtered (< 1 expected error per read) and gene-specific primers were trimmed using *skewer*^26^. Next, we denoised the fastq files using *dada2*^27^ to obtain 16S rRNA haplotypes (hereafter OTUs). Chimeric OTUs were eliminated by *uchime*^28^ and taxonomy for non-chimeric OTUs was assigned using *RDP classifier* (> 80% confidence^29^) and the *Silva* database version 132^30^. BLCA^31^ was used in specific cases for species-level assignment. The OTU sequences were then aligned by *PyNAST*^32^ and their phylogeny reconstructed using *FastTree*^33^. PCR triplicates were prepared for each sample, all of them being sequenced twice on two Illumina MiSeq runs (i.e., six technical replicates per sample in total). We found high consistency in community profile composition among identical technical replicates between the two MiSeq runs (Mantel test: r = 0.991, p < 0.001), as well as between technical replicates within the first and second runs (Mantel test: r > 0.99, p < 0.001). To avoid microbial diversity inflation due to PCR and sequencing artifacts, we eliminated all OTUs that were present just once within the set of PCR replicates for a given sample, e.g.^34^. We also excluded OTUs that were not assigned to any bacterial phylum, or those that were of chloroplast or mitochondrial origin. Furthermore, using *Decontam*^35^, we eliminated 82 OTUs corresponding to putative contaminants. The resulting abundance matrix, sample metadata, taxonomic assignations, haplotype sequences and their phylogeny were merged in a single phyloseq database^36^ for further analysis. The final dataset included 6,618,602 high-quality reads that passed all quality-filtering steps. Median sequencing coverage was 40,818 sequences per sample (range = 1,093–70,416).

### Statistical analysis

Shannon indices and number of OTUs were calculated after dataset normalization by rarefaction and used as alpha diversity measures. Variation in alpha diversity between species, between oral vs. vaginal samples, and during the course of captivity exposure was assessed using Analysis of variance (ANOVA) or Linear mixed effect models (LMM; *lmer* function in the R package *lme4*). If necessary, individual identity was included as a random effect in the LMM to account for repeated sampling of the same mouse. Box–Cox transformation of the alpha diversity indices was applied in order to achieve a normal distribution of ANOVA/LMM residuals. Variation in microbiota composition was examined using Principal Coordinate Analysis (PCoA) and PERMANOVA (adonis2 function in the R package *vegan*) running on relative abundance-based (Bray–Curtis) and prevalence-based (binary Jaccard) dissimilarities. To account for variation in sequencing depth, OTU counts were transformed to proportions prior to calculation of Bray–Curtis dissimilarities, while Jaccard dissimilarities were calculated after OTU table rarefaction. Individual identity was specified as a constraint for permutations (aka “strata”) if the PERMANOVA model included multiple samples from the same individual. We first tested the effect of species identity and sample type, after which separate PERMANOVA analyses for the oral and vaginal data subsets were conducted to assess the effect of captive conditions and species identity on microbiota changes and the effect of sex on oral microbiota composition. Below, we report marginal probability values (i.e., the significance of a predictor controlled for the effects of all other explanatory variables in the given model). In parallel to the PERMANOVA analyses, we searched for specific OTUs where changes in abundance played a role in the compositional variation, using Joint Species Distribution Models (JSDM) from the package *boral*^37^. For the JDSM, we assumed a negative binomial distribution of OTU counts and only considered OTUs present in > 5 samples. Individual identity was modeled via random effects and log-scaled sequencing coverage per sample as a model offset. Explanatory variables in the JSDM were the same as those included in the corresponding PERMANOVA tests. Two latent variables were estimated during the fitting process to account for residual correlation of OTU abundances. JSDM coefficients were estimated via 50,000 iterations of a Markov Chain Monte Carlo simulation using default priors. The thinning interval was set to 20 iterations and the first 50 iterations were discharged as a burn in. Support for the estimated parameters was assessed based on 95% posterior credible intervals. We also applied phylogenetic placement analysis for selected OTUs by extracting all reference 16S rRNA sequences corresponding to the same genus as the OTUs in question from the Silva database and clustering them at 99% similarity using *vsearch*^38^. Representative sequences for clusters exhibiting > 97% sequence similarity with any OTU were used for phylogenetic reconstruction using *RAxML*^39^, assuming the GTRI substitution model after *mafft* alignment^40^. Bootstrap analysis (1,000 replicates) was conducted to assess the robustness of phylogenetic clades. All the figures were generated in R software (https://cran.r-project.org/).

### Ethical standards

All animal procedures were carried out in strict accordance with the law of the Czech Republic paragraph 17 no. 246/1992. This study was, in accordance with accreditation no. 27335/2013-1721, approved by local ethics committee of the Faculty of Science, Charles University in Prague chaired by Stanislav Vybíral, Ph.D.

## Results

### Oral and vaginal microbiota in wild *Apodemus* populations

**Figure 1:**
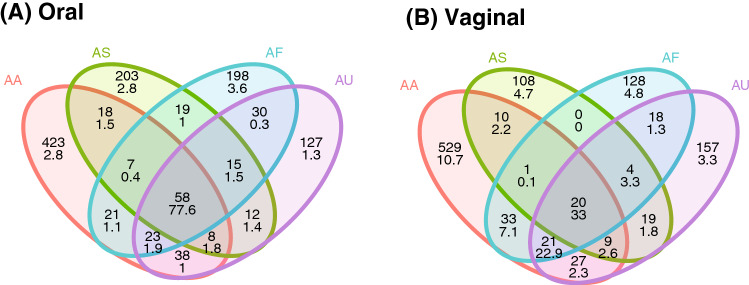
Venn diagrams for number of bacterial OTUs (upper numbers) and corresponding proportions of 16S rRNA reads (lower numbers) in (**A**) oral and (**B**) vaginal microbiota shared among four free-living *Apodemus* species (AA—*A. agrarius*, AF—*A. flavicollis*, AS—*A. sylvaticus*, AU—*A. uralensis*).

We detected 1,084 vaginal OTUs and 1,200 oral OTUs in wild populations, most of which were unique for individual *Apodemus* species, only a limited fraction (58 oral OTUs and 20 vaginal OTUs) being shared by all four species. Oral OTUs shared interspecifically corresponded to the dominant bacteria and were represented by 77% of all reads from the oral profiles. On the contrary, only 33% of vaginal reads represented vaginal OTUs shared by all species (Fig. 1).

**Figure 2:**
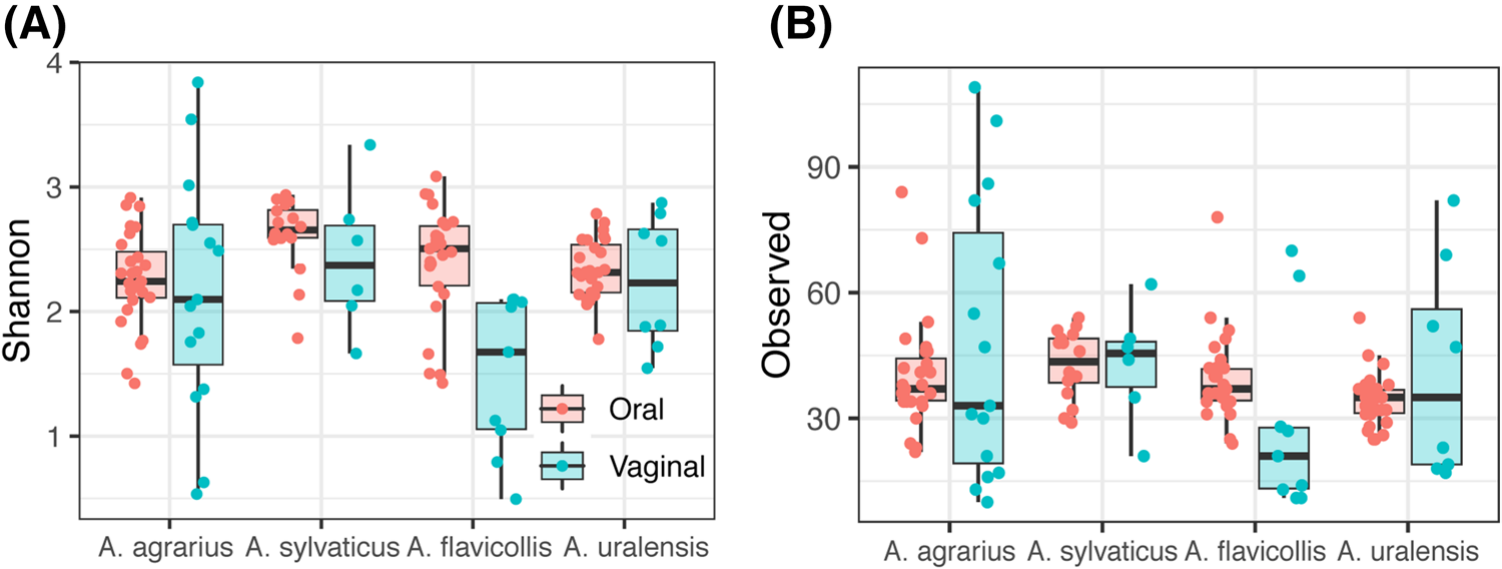
Boxplots for Shannon diversity (**A**) and observed number of OTUs (i.e., 16S rRNA haplotypes) (**B**) in oral and vaginal microbiota of each *Apodemus* species. Both Shannon diversities and OTU richness were calculated using rarefied OTU table.

We found no correlation in alpha diversity between vaginal vs. oral microbiota sampled from the same individual (Pearson correlation: d.f. = 35, r = 0.0010, p = 0.9952 for Shannon diversities and r = 0.2672, p = 0.1098 for number of OTUs). Oral communities more diverse according to Shannon indices (LME: ΔDF = 3, χ^2^ = 7.9812, p = 0.0464), but did not harbor more OTUs (LME: ΔDF = 3, χ^2^ = 3.2051, p = 0.0734). At the same time, the difference between these two sample types was not the same across the four *Apodemus* species, as indicated by species × sample type interaction (LME: ΔDF = 3, χ^2^ = 13.031, p = 0.0046 for Shannon diversity and ΔDF = 3, χ^2^ = 8.866, p = 0.0311 number of OTUs). The most pronounced difference in diversity between oral and vaginal microbiota was observed in *A. flavicollis.* When focusing on the subset of vaginal samples, we detected no interspecific variation in diversity (ANOVA: F_(3,34)_ = 2.299, p = 0.0949 for Shannon diversity and F_(3,34)_ = 1.388, p = 0.2632 for number of OTUs) (Fig. 2). In the case of oral samples, *A. sylvaticus* exhibited the most diverse microbiota and *A. uralensis* the least (p = 0.0314 for Shannon index and p = 0.0090 for number of OTUs according to Tukey post-hoc tests). Further, while the Shannon diversity of *A. agrarius* oral microbiota was significantly lower than that of *A. sylvaticus* (p = 0.0165), a similar contrast in the number of observed OTUs was not significant (p = 0.6683). In addition, male’s Shannon diversity of oral microbiota was significantly decreased (F_(1,90)_ = 10.972, p = 0.0013) they also tended to harbor a lower number of OTUs in oral microbiota than females (F_(1,90)_ = 3.875, p = 0.0521).

**Figure 3:**
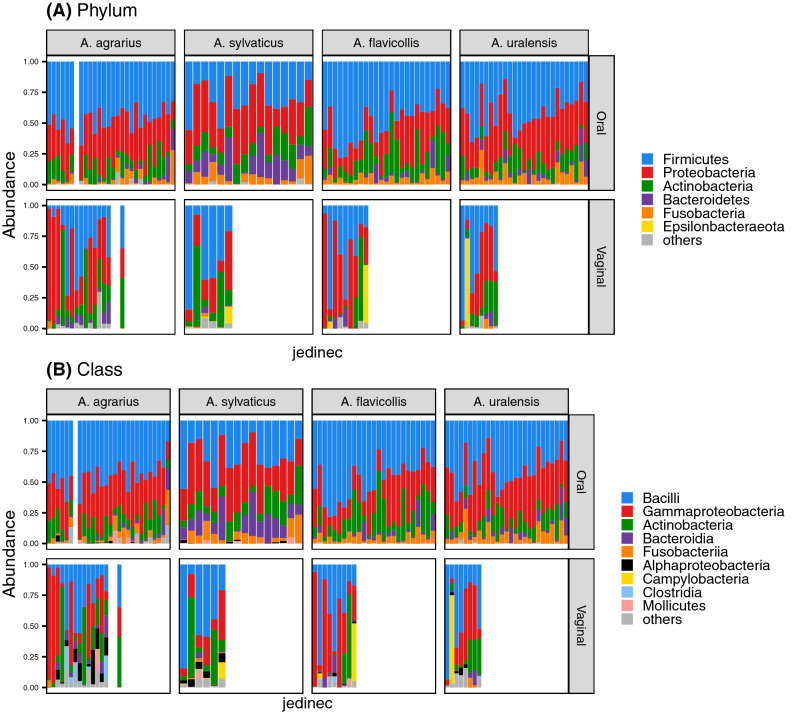
Relative abundance of dominating (i.e., represented by > 1% of reads) bacterial (**A**) phyla and (**B**) classes in oral and vaginal microbiota of *Apodemus* species sampled in free-living populations.

Irrespective of species identity, vaginal and oral microbiota exhibited systematic differences in composition (Figure S1, PERMANOVA: pseudo-F_(1,128)_ = 18.129, p < 0.001 for Bray–Curtis and pseudo-F_(1,128)_ = 9.985, p < 0.001 for Jaccard dissimilarities). According to taxonomic barplots (Fig. 3), Bacilli, Gammaproteobacteria, and Actinobacteria were the dominant bacterial classes in both oral and vaginal samples. Fusobacteria were present in most oral samples, while their occurrence in vaginal microbiota was rather rare. On the other hand, Alphaproteobacteria were prevalent in vaginal microbiota samples but not in oral. JSDM controlled for within-individual covariance and systematic interspecific differences in microbiota composition identified 122 OTUs whose abundance varied between oral and vaginal microbiota (Figure S2). Despite the overall consistency of JSDM with the taxonomic barplots, JSDM also suggested considerable differentiation between oral and vaginal microbiota at a relatively small taxonomic scale. For example, most OTUs from the genera *Streptococcus, Lactobacillus,* unassigned *Carnobacteriaceae, Rodentibacter*, and unassigned *Pasteurellaceae* were more abundant in oral samples, while just a few OTUs belonging to these genera were more prevalent in vaginal microbiot***a (Figure S2).

There was a higher compositional similarity between oral and vaginal profiles from the same individual than expected by chance (Procrustes analysis: sum of squares = 0.126, r = 0.935, p = 0.001 for Jaccard and sum of squares = 0.573, r = 0.653, p = 0.009 for Bray–Curtis dissimilarities). However, if we specified species identity as a blocking factor for permutation testing, the correlation for Bray–Curtis dissimilarity lost its significance (p = 0.136) and was only marginally non-significant in the case of Jaccard dissimilarity (p = 0.054). We conclude, therefore, that similarity pattern across the two body regions was driven more by a correlated interspecific divergence of oral and vaginal communities than by within-species effects.

**Figure 4:**
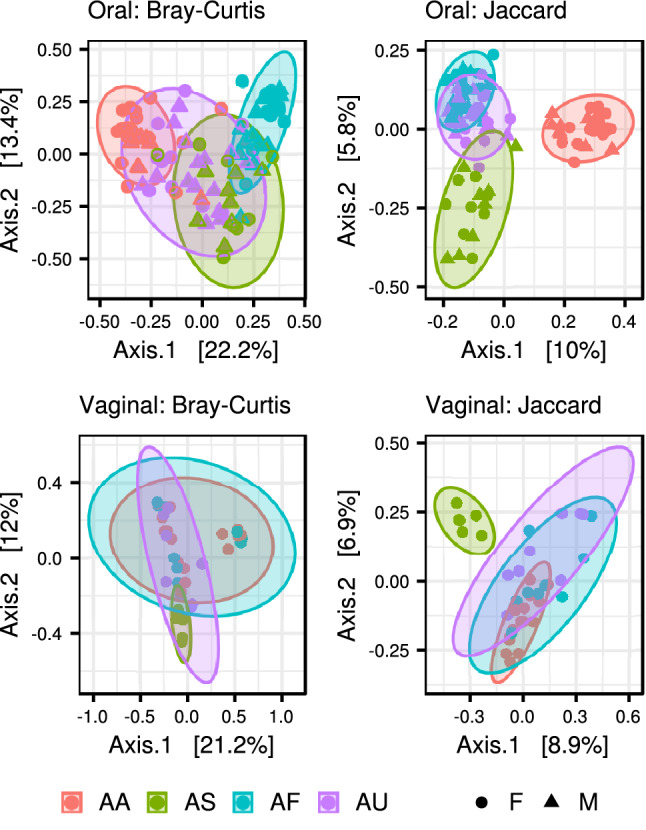
PCoA for oral and vaginal microbiota from four free-living *Apodemus* species (AA—*A. agrarius*, AF—*A. flavicollis*, AS—*A. sylvaticus*, AU—*A. uralensis*). Ordination was conducted for dissimilarities accounting for OTU absence vs. presence (Jaccard) and relative abundance (Bray–Curtis). Standard 95% ellipses are shown to highlight the ordination space for each species.

While oral microbiota composition exhibited considerable interspecific differences (PERMANOVA: pseudo-F_(3,90)_ = 11.882, p = 0.001 for Bray–Curtis and (pseudo-F_(3,90)_ = 6.325, p = 0.001 for Jaccard dissimilarities), sex had no effect (pseudo-F_(1,90)_ = 1.670, p = 0.060 for Bray–Curtis and pseudo-F_(1,90)_ = 1.025, p = 0.416 for Jaccard dissimilarities. PCoA for prevalence-based dissimilarities showed that oral samples of *A. agrarius* and *A. sylvaticus* formed separate clusters exhibiting minimal overlap with the other two species. In comparison, there was considerable overlap in ordination space across all species according to abundance-based dissimilarities (i.e. Bray–Curtis) with *A. flavicollis* exhibiting the most distinct composition compared to the other two species (Fig. 4). According to betadisper analysis, interindividual variation in microbiota composition was not constant at the interspecific level, though the overall pattern strongly depended on actual dissimilarity measure (Figure S3).

Unlike oral microbiota, the ordination space occupied by vaginal samples from free-living *Apodemus* species exhibited pronounced overlap in both prevalence- and abundance-based dissimilarities (Fig. 4). Nevertheless, PERMANOVA revealed significant differences in vaginal microbiota composition at the interspecific level (F_(3,34)_ = 2.018, p = 0.003 for Bray–Curtis dissimilarities and F_(3,34)_ = 2.042, p = 0.001 for Jaccard dissimilarities). According to betadisper analysis for OTU prevalence, interindividual variation was constrained in *A. sylvaticus* and *A. uralensis* compared to the other two species. A similar analysis based on OTU abundance also found non-significantly constrained interindividual variation in *A. sylvaticus* microbiota (Figure S3). JSDMs identified 84 OTUs in oral microbiota whose abundances varied between species (Figures S4 and S5).

### Effect of captivity on oral and vaginal microbiota

**Figure 5:**
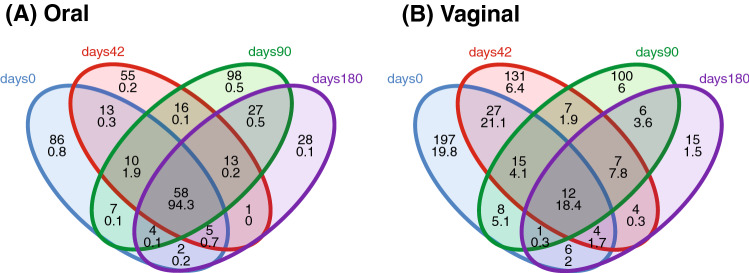
Venn diagrams for the number of bacterial OTUs (upper numbers) and corresponding proportions of 16S rRNA reads (lower numbers) in (**A**) oral and (**B**) vaginal microbiota of *A. uralensis* following its introduction to captive conditions.

A relatively large number of OTUs from both vaginal and oral microbiota were exclusive for each of the sampling periods (Fig. 5), with only 12 vaginal and 58 oral OTUs persisting across all sampling phases. However, persisting oral OTUs represented 94% of all oral microbiota reads in captive individuals, while only 18% of vaginal OTU reads persisted throughout captivity exposure. Interestingly, most of the oral OTUs persisting throughout the period of captivity were shared by all four free-living *Apodemus* species (n = 34). This suggests the existence of a conserved set of core oral microbiota OTUs (Table S1) and, at the same time, suggests a relatively dramatic turnover of vaginal microbiota over time due to environmental changes.

**Figure 6:**
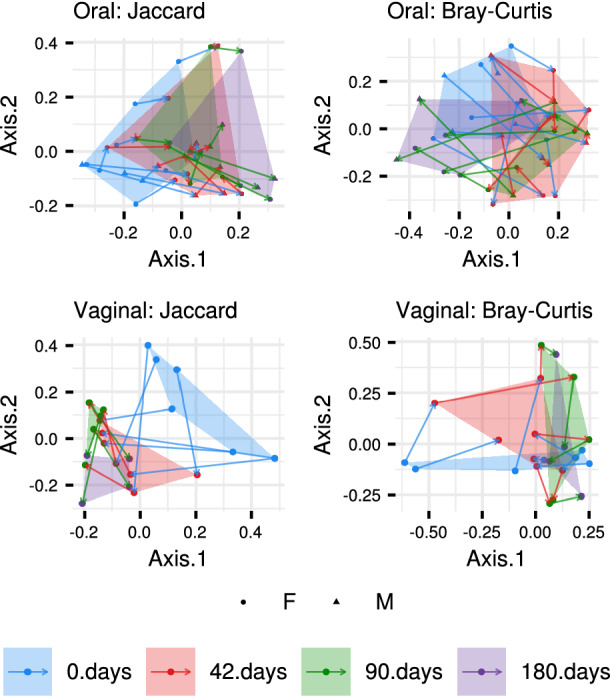
PCoA describing compositional changes in oral and vaginal microbiota of *A. uralensis* following its introduction to captive conditions. Arrows connect microbiota samples individual mice. Ordination was conducted for dissimilarities accounting for OTU absence vs. presence (Jaccard) and relative abundance (Bray–Curtis). Standard 95% ellipses are shown to highlight the ordination space for each species.

**Figure 7:**
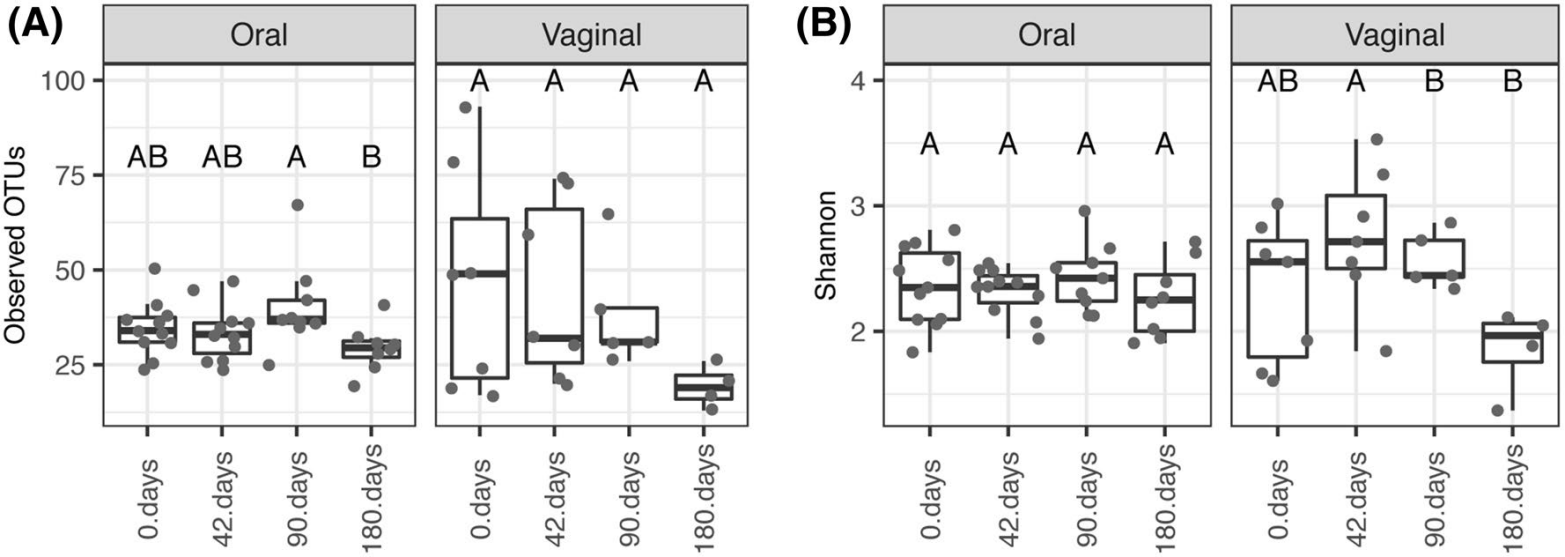
Alpha diversity changes in *A. uralensis* oral and vaginal microbiota following exposure to captive conditions. Observed number of OTUs (**A**) and the Shannon index (**B**) were used as alpha diversity measures.

This conclusion is in line with the more pronounced gradual changes in vaginal OTU prevalence over the course of captivity (Fig. 6; PERMANOVA: pseudo-F_(3,19)_ = 1.7205, p < 0.0001) compared to changes observed in oral microbiota (PERMANOVA: pseudo-F_(3,34)_ = 1.5898, p < 0.0001). Nevertheless, the same analysis comparing temporal variation in OTU abundance revealed large overlaps between samples from different treatment phases, with no apparent hint of gradual shifts in microbiota composition (Fig. 6; pseudo-F_(3,34)_ = 3.3949, p < 0.0001 for vaginal and pseudo-F_(3,19)_ = 1.4118, p = 0.012 for oral microbiota). Consistent with the increased robustness of oral microbiota to changes in environmental conditions induced by captivity, there was no temporal variation in Shannon diversity in oral profiles (ΔDF = 3, χ^2^ = 1.8507, p = 0.604; Fig. 7) or number of observed OTUs exhibiting a slight increase on the 90th day when compared with the 180th day (ΔDF = 3, χ^2^ = 8.1004, p = 0.04398, Tukey post-hoc test: p = 0.0199). On the other hand, Shannon diversity on vaginal microbiota exhibited a non-significant increase during the first sampling after captivity exposure (i.e., 42nd day), followed by a decrease in diversity between the 42nd vs. 180th and 90th vs. 180th days of captivity (ΔDF = 3, χ^2^ = 11.756, p = 0.008267, Tukey post-hoc test: p < 0.05).

## Discussion

Microbial communities have considerable effects on the fitness-related phenotype traits of their hosts^3^, which makes them indisputable components of current evolutionary theory^41^. However, most of the research effort to date has been dedicated solely to bacterial communities residing in the gut. Consequently, there is still a scarcity of information on the composition, within- and between-species variation, and phenotype effects of microbiota associated with other organs. Here, we investigated how oral and vaginal microbiota differed among four free-living murine species (genus *Apodemus*) with differing behavioral systems using high-throughput 16S rRNA amplicon sequencing. To test microbiota robustness against environmental change, we also introduced one of the species (*A. uralensis*) into captivity and characterized shifts in oral and vaginal microbiota over time.

Females' oral and vaginal microbiotas can be potentially regularly intermingled via genital self-grooming^42^. Despite the fact that both oral and vaginal samples were dominated by bacteria from the phyla Firmicutes (with *Lactobacillus*, *Streptococcus*, and *Staphylococcus* the most abundant genera), Proteobacteria (especially *Rodentibacter*) and Actinobacteria (especially *Corynebacteriaceae*), we observed striking compositional divergence between the two microbiotas. This was even more decisive than host species identity which suggests, congruently with published data^43^, that the body site is a very strong influential factor in terms of microbiota composition.

While several recent studies have characterized oral or vaginal microbiota in captive mammals^44–46^, these communities are rarely scrutinized in wild populations. As such, our findings extend those of the only existing study on oral microbiota in a free-living murine species (the house mouse *Mus musculus musculus*^47^), for which dominance of *Pasteurellaceae* of the phylum Proteobacteria (> 75%) was reported. In *Apodemus* mice, *Pasteurellaceae* (represented mainly by *Rodentibacter*) comprised a considerable fraction of the oral microbiota, though its percentage was lower compared to that of the house mouse (~ 25% of sample reads). A number of recent studies have also investigated the vaginal microbiota in non-human, mostly captive mammals^48–50^. Consistent with these studies, our data revealed that, unlike the *Lactobacillus*-dominated community in humans^5^, the vaginas of non-human mammals harbor a taxonomically more diverse spectrum of bacteria. In the case of free-living *Apodemus* mice, these include mainly *Corynebacterium*, *Rodentibacter*, *Campylobacter*, and *Staphylococcus*. Nevertheless, the 19% of lactobacilli (represented mainly by *L. reuteri*, *L. intestinalis*, *L. johnsonii*, *L. gasseri* and *L. murinus*, Figure S6) recorded on average in *Apodemus* vaginal samples was still relatively high compared to most other non-human mammals (reviewed in^51^).

Both oral and vaginal microbiota exhibited a significant level of interspecific variation, with the strength of between-species divergence being more pronounced in the case of oral communities. Partial overlaps in composition between different species could be explained by the relatively low number of highly abundant OTUs shared by all *Apodemus* species. These represented up to 78% of all sequences in oral samples and, surprisingly, most of them persisted in *A. uralensis* throughout the captivity exposure experiment. Moreover, we observed only slight changes in alpha diversity and a slow pace of shifts in OTU prevalence at the whole community level. These findings imply the existence of a set of “core” oral bacteria exhibiting a conserved association with the host across speciation events and persistence during changes in environmental conditions. In line with previous research, we propose that the set of core bacteria may include keystone species that are involved in indispensable metabolic pathways^52^, the depletion of which may disturb ecosystem services provided by microbiota to the host and result in detrimental effects on host fitness. Indeed, the human oral microbiota works as a crucial component of the ecosystem stability by excluding the pathogens together with contribution to normal development of tissue and immune system^53,54^.

Contrary to oral microbiota, vaginal OTUs shared by all species constituted a much lower fraction of the whole community, with just a few being consistently detected throughout the period of captivity treatment. During captivity, dramatic variation in vaginal microbiota diversity and rapid changes in OTU prevalence were observed at the whole community level. This implies higher plasticity of vaginal microbiota over time and/or changes in response to altered environmental conditions. This is surprising, given the well-recognized role of vaginal microbiota in defense against pathogens in humans^55,56^ and given that changes induced by captivity, such as diet and restriction of social contact with other population members.

The above-described contrasts in the structure and variation of oral and vaginal communities suggest different mechanisms behind their interspecific divergence. Ecological and phylogenetic effects have been shown to play critical roles in between-species variation in gut microbiota^19,57^. At the same time, social interactions have been shown to facilitate horizontal and vertical microbiota transfer and are thus recognized as another significant source of microbiota variation at the intraspecific level^58,59^. Nevertheless, it remains to be answered whether variation in social behavior affects the divergence of microbiota structure between species. To the best of our knowledge, there has been just one study, based on a comparison of two deer mice species (genus *Peromyscus*), examining whether there is any association between interspecific differences in promiscuity and vaginal microbiota diversity^60^ Here, we predicted that interspecies changes in social and mating behavior could explain differences in *Apodemus* oral and vaginal microbiota. We used previously published information on the frequency of multiple male mating as a proxy for variation in the mating system^20^ and compared this with our data on interspecific variation in microbiota diversity patterns. However, this comparison provided no conclusive support for the effect of mating behavior on microbiota structure. Specifically, interspecific variation in alpha diversity did not follow the prediction that more promiscuous species and/or those exposed to more frequent social interaction harbor more diverse microbial communities. First, there were no significant interspecific differences in vaginal microbiota diversity and the species with the least diverse vaginal microbiota (*A. flavicollis*) exhibited intermediate frequency of multiple-male mating. Second, the species with the highest level of multiple-male mating (*A. sylvaticus*) exhibited the highest oral microbiota diversity. Nevertheless, *A. agrarius*, another highly-promiscuous species, exhibited comparable diversity to the least promiscuous species (*A. uralensis*). We also expected that differences in mating and social behavior could affect inter-individual variation of microbiota composition due to differing intensity of microbiota transfer through social contact. This prediction does not hold, however, as the highest and lowest interindividual variation in vaginal microbiota was detected in *A. agrarius* and *A. sylvaticus*, respectively; both being species with high frequencies of multiple male mating. Furthermore, interindividual variation of oral microbiota did not exhibit consistent interspecific pattern independent of analytical tools.

One of the most influential environmental factor—diet—was not examined although it might contribute to the interspecific divergence of oral microbiota. However, several studies support the hypothesis that the Apodemus spp. overlap in their ecological niches and even compete among each other for the food sources (i.e. seed and fruit of trees, invertebrates)^61,62^. However, for the future studies it would be interesting to scrutinize the content of the intestine/feces to have a knowledge about food preferences for each individual.

Collectively, this work contributes to microbiota research in two major aspects. Firstly, it extends the few studies focused on non-human oral and vaginal microbiota, and secondly, by examining wild rodents and those transferred to captivity, we confirmed that each microbiota responds differently to a changing environment. Our results could help in the detection of unique features in both oral and vaginal microbiota, groups that are often explored in relationship to different diseases^5,46^.

## Supplementary information

Supplementary Information.

## Data Availability

Sequencing data for individual samples will be available from the European Nucleotide Archive.
