# Supplementary material for: Oral and vaginal microbiota in selected field mice of the genus Apodemus: a wild population study
Source: Sci Rep. 2020 Aug 6;10:13246. doi: 10.1038/s41598-020-70249-x (PMC7413396; doi:10.1038/s41598-020-70249-x)
Supplement: Supplementary file 1 — Supplementary Information. [file 41598_2020_70249_MOESM1_ESM.pdf]

Oral and vaginal microbiota in selected field mice of the genus *Apodemus*: a wild population study

Tereza Matějková[1] , Petra Hájková[1,2], Romana Stopková[1], Michal Stanko[3], Jean-François Martin[4] , Jakub Kreisinger[1]†, Pavel Stopka[1]†

[1] *Department of Zoology, Faculty of Science, Charles University, BIOCEV, Vestec, Czech Republic*
[2] *Department of Population Biology, Institute of Vertebrate Biology, Academy of Sciences of the Czech Republic, Brno, Czech Republic*
[3] *Institute of Parasitology, Slovak Academy of Sciences, Slovakia*
[4] *Montpellier-SupAgro, UMR Centre de Biologie pour la Gestion des Populations, Montferrier-sur-Lez, France*
† These authors contributed equally.

[*] Correspondence: Pavel Stopka, pstopka@natur.cuni.cz; Jakub Kreisinger, jakubkreisinger@seznam.cz

SUPPLEMENTARY FIGURES:

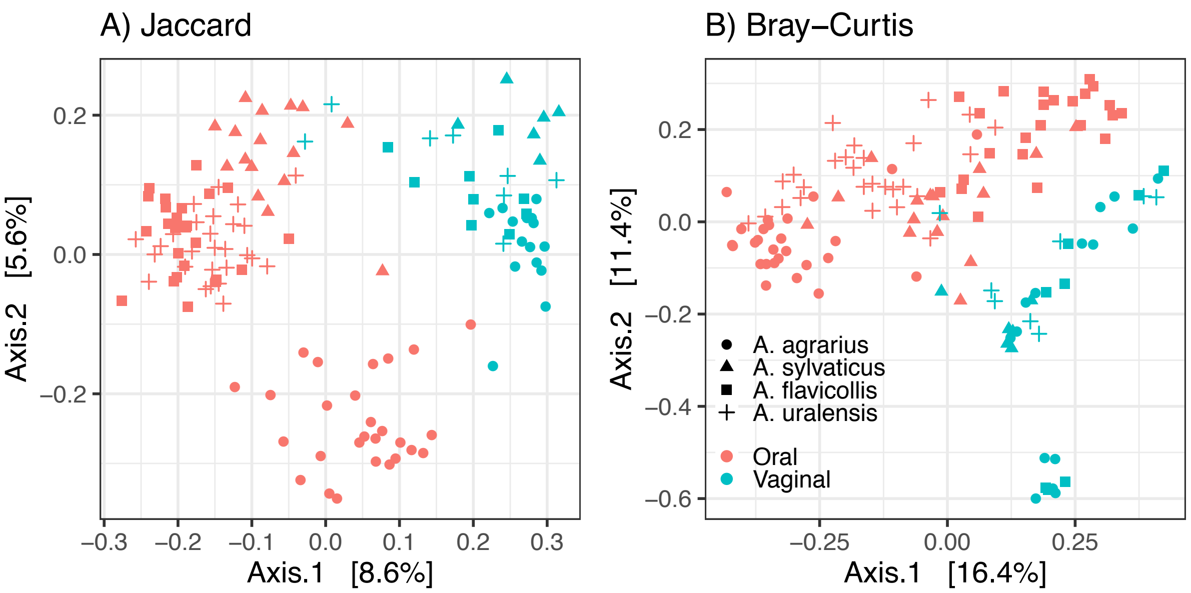

Figure S1: PCoA ordination for compositional variation in oral and vaginal microbiota of four free-living Apodemus species. Ordination was conducted for dissimilarities accounting for OTU absence vs. presence (Jaccard) (A) and relative abundance (Bray-Curtis) (B).

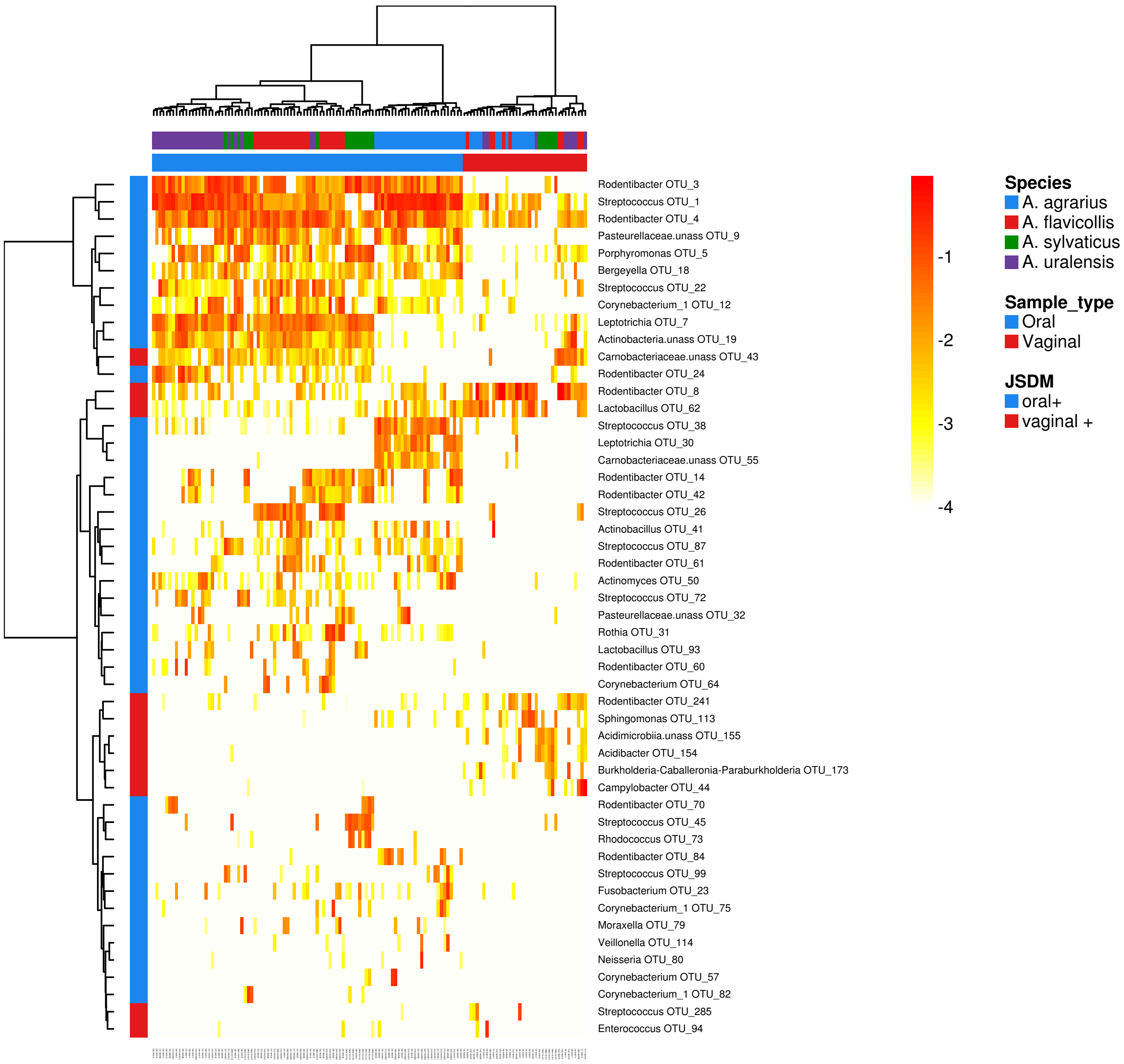

Figure S2: Heatmaps for proportions (log scaled) of the 50 most abundant OTUs whose abundance varied between oral and vaginal microbiota according to JSDM analysis. Column annotations indicate species identity and microbiota class (i.e., oral vs. vaginal). Row annotations indicate over- and under-representation of a given OTU in the oral and vaginal subsets. The matrix was sorted using Ward's method.

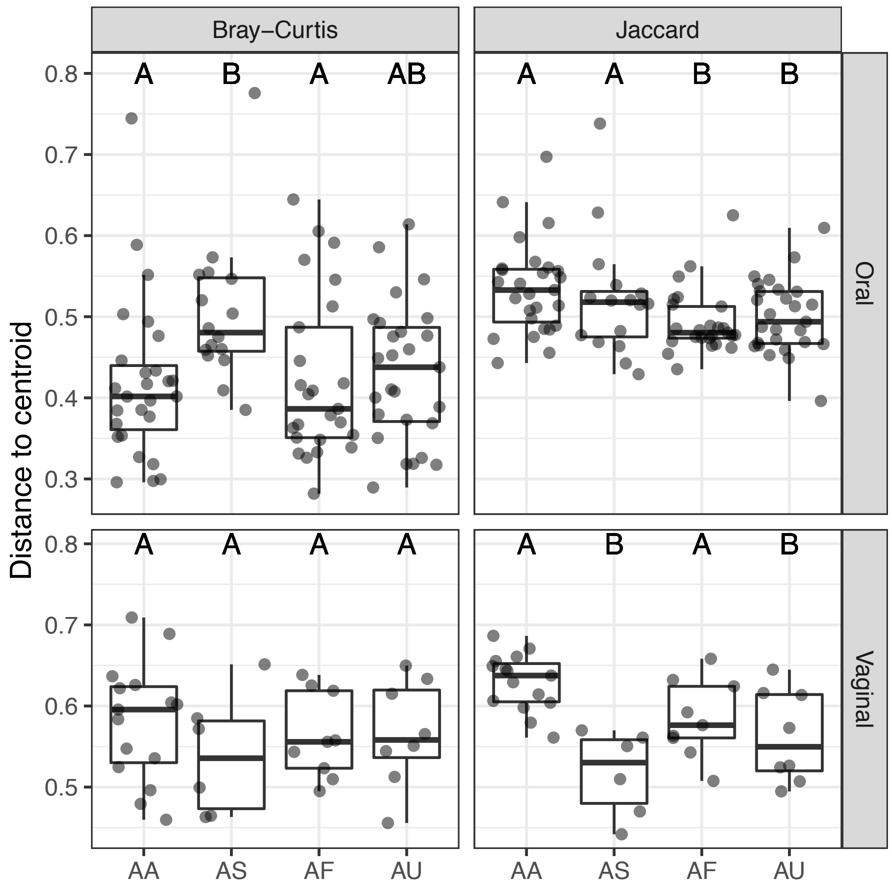

Figure S3: Inter-individual variation of oral and vaginal microbiota for four *Apodemus* species, assessed as the distance to the centroid. Interspecific differenceswere analyzed by betadisper. Different letters above the box plots indicate significant differences according to Tukey post-hoc tests.

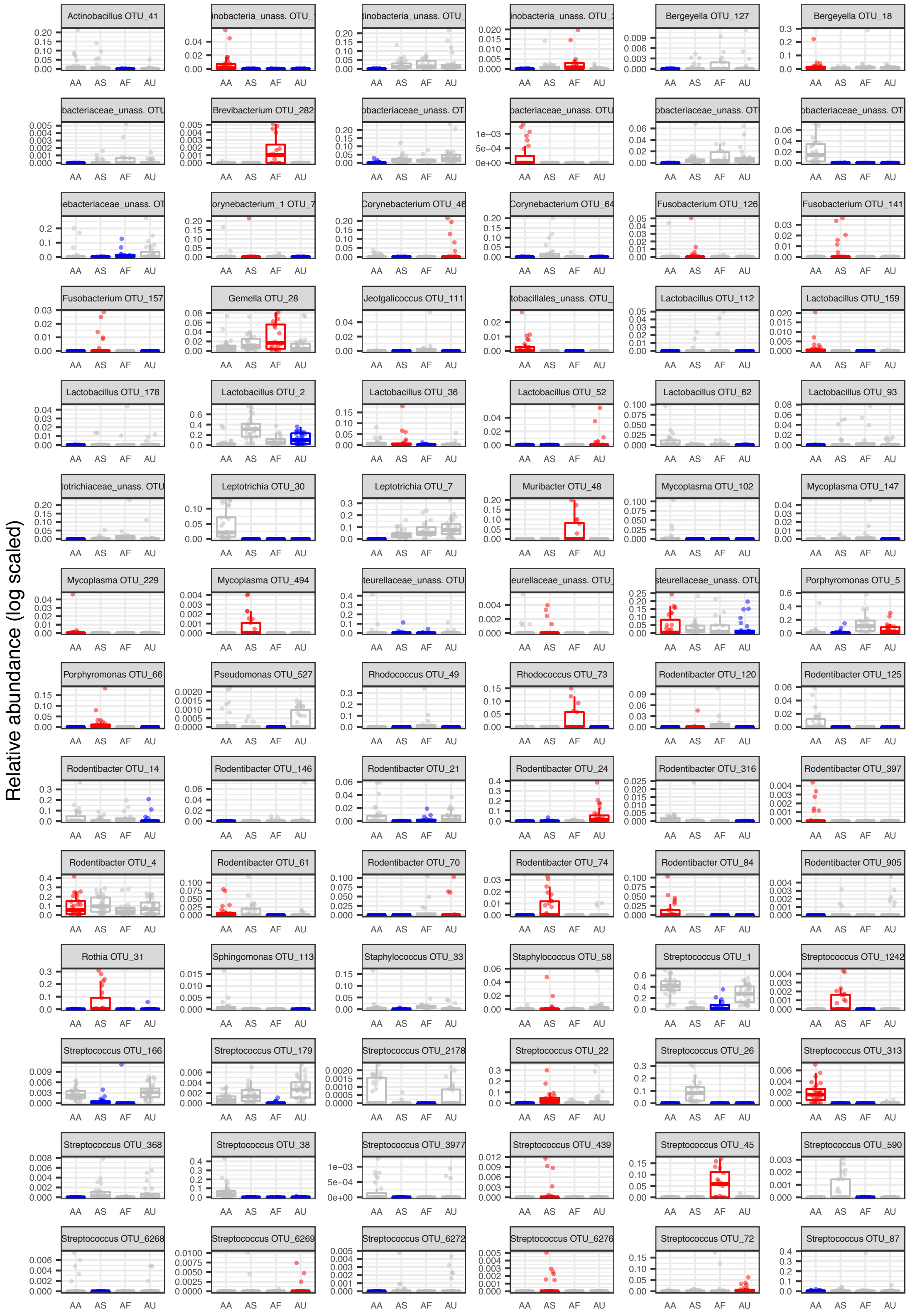

Figure S4: Boxplots for oral OTUs whose abundances varied between four Apodemus species (AA – *A. agrarius*, AF – *A. flavicollis*, AS – *A. sylvaticus*, AU – *A. uralensis*) according to JSDM. A significant increase in abundance in a given species (compared to the average) is indicated in red, whereas a significant decrease is indicated in blue.

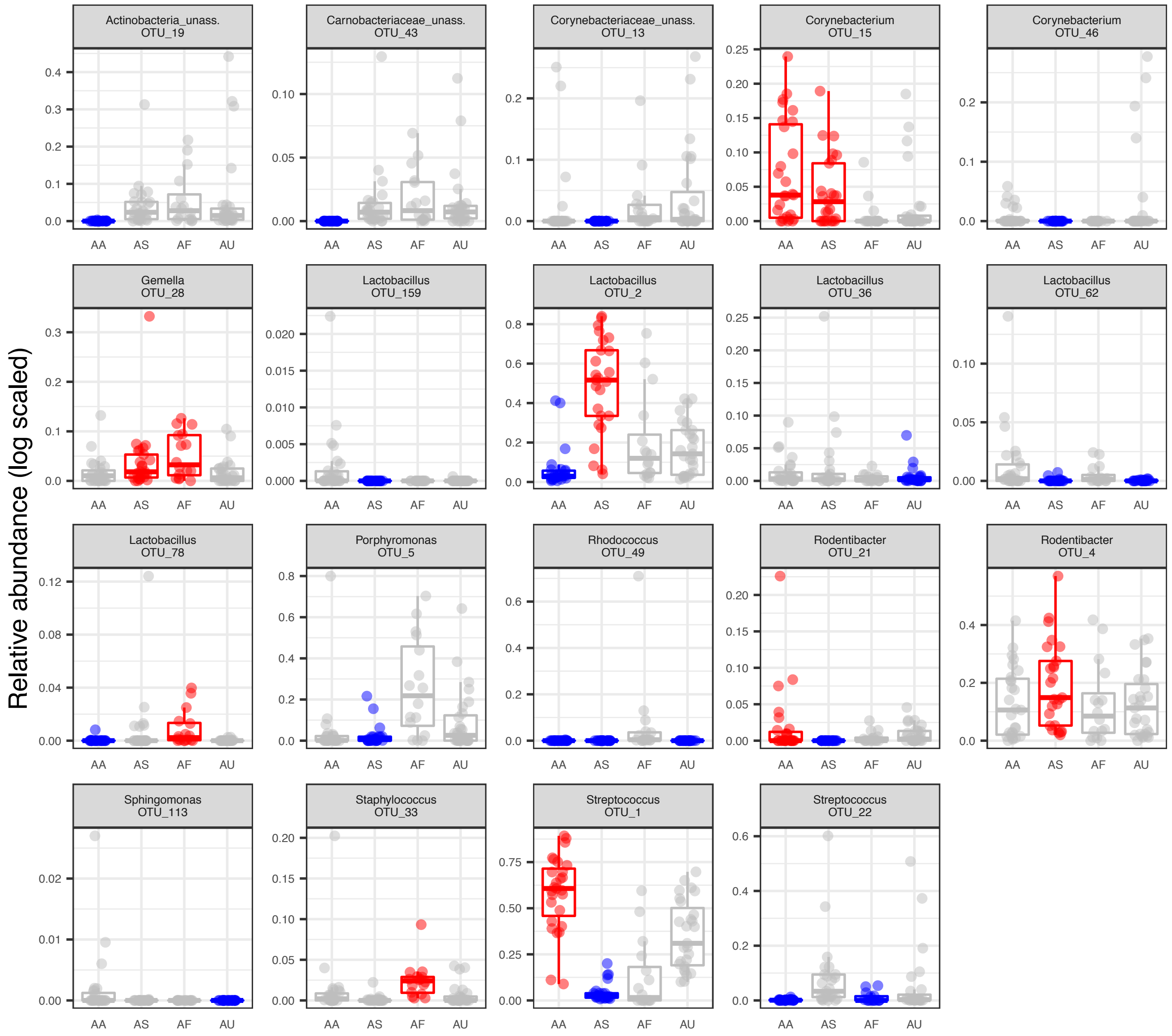

Figure S5: Boxplots for bacterial OTUs whose abundances in vaginal microbiota varied between species according to JSDM. A significant increase in abundance in a given species (compared to the average) is indicated in red, whereas a significant decrease is indicated in blue colors.

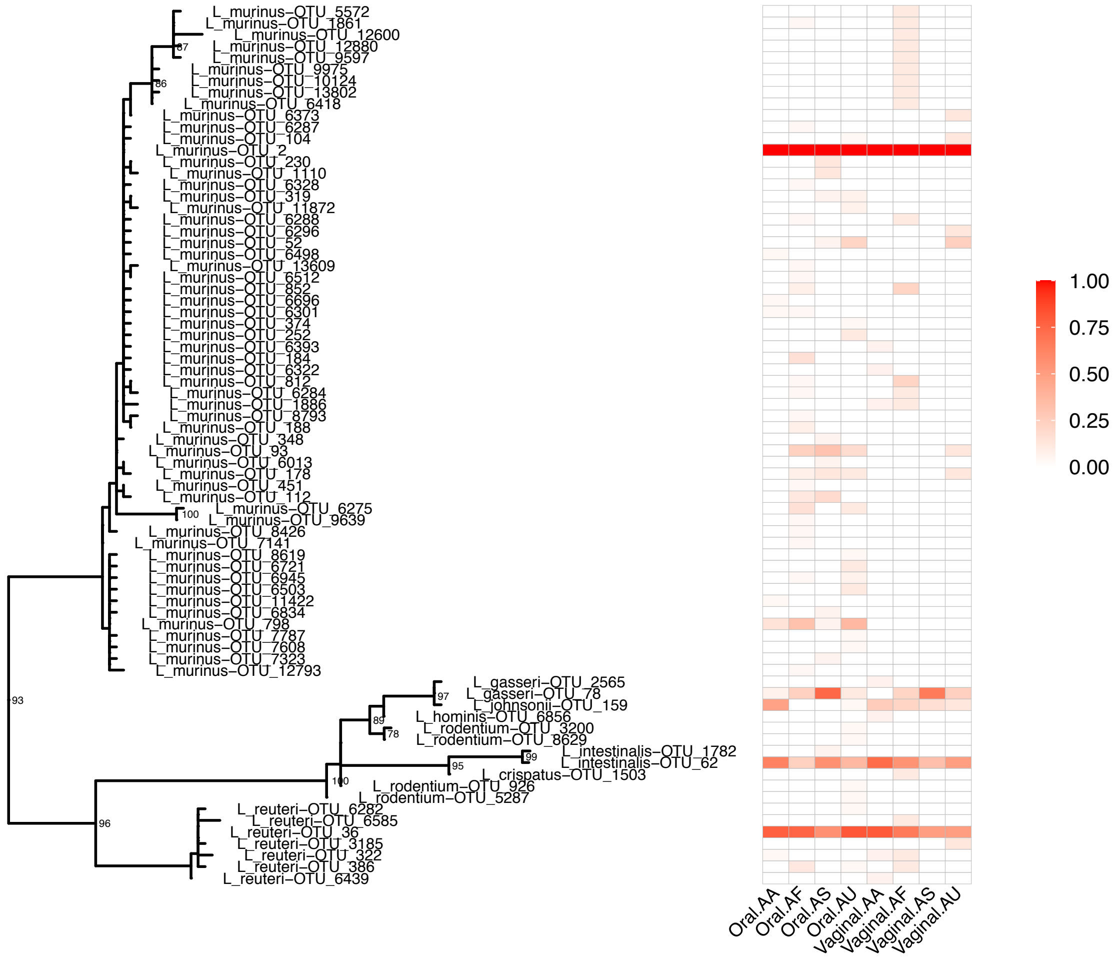

Figure S6: Prevalences of Lactobacillus OTUs detected in oral and vaginal microbiota of four *Apodemus* species. Species-level assignation was conducted using BLCA software.

Table S1: Core microbiota of *Apodemus* mice. The list shows oral and vaginal OTUs that were detected in all four free-living *Apodemus* species and/or that persisted in the captive A. uralensis population throughout the experimental period. Relative abundances are shown along with taxonomic assignments up to genus level. Empty cells indicate that a given OTU was not detected in all free-living species or that it did not persist in the captive population.

Microbiome	OTU	Class	Genus	rel.abund.c	rel.abund.w
Oral	OTU_1	Bacilli	Streptococcus	0.1567	0.154
	OTU_2	Bacilli	Lactobacillus	0.1061	0.1143
	OTU_3	Gammaproteobacteria	Rodentibacter	0.0998	0.0854
	OTU_4	Gammaproteobacteria	Rodentibacter	0.1006	0.0724
	OTU_5	Bacteroidia	Porphyromonas	0.0421	0.036
	OTU_7	Fusobacteriia	Leptotrichia	0.0383	0.0364
	OTU_9	Gammaproteobacteria	Pasteurellaceae_unass.	0.0432	0.0251
	OTU_16	Bacilli	Gemella	0.0509	5.00E-04
	OTU_14	Gammaproteobacteria	Rodentibacter	0.024	0.0188
	OTU_50	Actinobacteria	Actinomyces	0.0356	0.0066
	OTU_12	Actinobacteria	Corynebacterium_1	0.0121	0.0228
	OTU_15	Actinobacteria	Corynebacterium	0.0092	0.0186
	OTU_22	Bacilli	Streptococcus	0.0094	0.016
	OTU_18	Bacteroidia	Bergeyella	0.0167	0.0081
	OTU_19	Actinobacteria	Actinobacteria_unass.	0.01	0.0138
	OTU_23	Fusobacteriia	Fusobacterium	0.0181	0.0042
	OTU_25	Bacilli	Carnobacteriaceae_unass.	0.0063	0.0135
	OTU_32	Gammaproteobacteria	Pasteurellaceae_unass.	0.0128	0.0068
	OTU_28	Bacilli	Gemella	0.0057	0.0114
	OTU_38	Bacilli	Streptococcus	1.00E-04	0.0139
	OTU_36	Bacilli	Lactobacillus	0.0046	0.0057
	OTU_42	Gammaproteobacteria	Rodentibacter	0.0012	0.0082
	OTU_72	Bacilli	Streptococcus	0.0052	0.0042
	OTU_33	Bacilli	Staphylococcus	0.001	0.0049
	OTU_8	Gammaproteobacteria	Rodentibacter	8.00E-04	0.0032
	OTU_58	Bacilli	Staphylococcus	0.0016	0.0015
	OTU_122	Gammaproteobacteria	Rodentibacter	0.0016	0.0014
	OTU_62	Bacilli	Lactobacillus	7.00E-04	0.0023
	OTU_78	Bacilli	Lactobacillus	0.001	0.0015
	OTU_116	Gammaproteobacteria	Rodentibacter	0.001	7.00E-04
	OTU_200	Gammaproteobacteria	Pasteurellaceae_unass.	7.00E-04	6.00E-04
	OTU_235	Gammaproteobacteria	Pasteurellaceae_unass.	5.00E-04	5.00E-04
	OTU_162	Bacilli	Streptococcus	3.00E-04	3.00E-04
	OTU_670	Gammaproteobacteria	Rodentibacter	2.00E-04	3.00E-04
	OTU_88	Gammaproteobacteria	Conchiformibius	0.0066	
	OTU_146	Gammaproteobacteria	Rodentibacter	0.0023	
	OTU_81	Gammaproteobacteria	Rodentibacter	0.0057	
	OTU_70	Gammaproteobacteria	Rodentibacter	0.002	

	OTU_60	Gammaproteobacteria	Rodentibacter	0.0098	
	OTU_1022	Gammaproteobacteria	Rodentibacter	1.00E-04	
	OTU_21	Gammaproteobacteria	Rodentibacter	0.0062	
	OTU_74	Gammaproteobacteria	Rodentibacter	0.0034	
	OTU_378	Gammaproteobacteria	Pasteurellaceae_unass.	5.00E-04	
	OTU_107	Gammaproteobacteria	Cardiobacteriaceae_unass.	0.0041	
	OTU_29	Bacteroidia	Porphyromonas	0.0289	
	OTU_91	Fusobacteriia	Leptotrichia	0.0044	
	OTU_95	Fusobacteriia	Leptotrichia	0.004	
	OTU_37	Fusobacteriia	Leptotrichiaceae_unass.	0.002	
	OTU_90	Negativicutes	Veillonella	0.0058	
	OTU_52	Bacilli	Lactobacillus	0.0126	
	OTU_6270	Bacilli	Streptococcus	5.00E-04	
	OTU_749	Bacilli	Streptococcus	4.00E-04	
	OTU_43	Bacilli	Carnobacteriaceae_unass.	0.0049	
	OTU_115	Bacilli	Gemella	0.0014	
	OTU_217	Bacilli	Gemella	0.0011	
	OTU_13	Actinobacteria	Corynebacteriaceae_unass.	0.0175	
	OTU_448	Actinobacteria	Bifidobacteriaceae_unass.	3.00E-04	
	OTU_203	Actinobacteria	Actinobacteria_unass.	2.00E-04	
	OTU_79	Gammaproteobacteria	Moraxella		0.0033
	OTU_41	Gammaproteobacteria	Actinobacillus		0.0085
	OTU_753	Gammaproteobacteria	Rodentibacter		1.00E-04
	OTU_453	Gammaproteobacteria	Rodentibacter		1.00E-04
	OTU_724	Gammaproteobacteria	Rodentibacter		2.00E-04
	OTU_673	Gammaproteobacteria	Rodentibacter		1.00E-04
	OTU_241	Gammaproteobacteria	Rodentibacter		1.00E-04
	OTU_24	Gammaproteobacteria	Rodentibacter		0.0137
	OTU_35	Gammaproteobacteria	Escherichia/Shigella		2.00E-04
	OTU_798	Bacilli	Lactobacillus		7.00E-04
	OTU_166	Bacilli	Streptococcus		0.0012
	OTU_179	Bacilli	Streptococcus		0.001
	OTU_889	Bacilli	Streptococcus		1.00E-04
	OTU_576	Bacilli	Streptococcus		2.00E-04
	OTU_87	Bacilli	Streptococcus		0.0035
	OTU_519	Bacilli	Streptococcus		1.00E-04
	OTU_99	Bacilli	Streptococcus		0.0035
	OTU_94	Bacilli	Enterococcus		0
	OTU_121	Bacilli	Staphylococcus		4.00E-04
	OTU_259	Bacilli	Staphylococcus		4.00E-04
	OTU_156	Bacilli	Staphylococcus		9.00E-04
	OTU_75	Actinobacteria	Corynebacterium_1		0.0043
	OTU_2667	Actinobacteria	Corynebacteriales_unass.		0
	OTU_31	Actinobacteria	Rothia		0.012
Vaginal	OTU_2	Bacilli	Lactobacillus	0.0823	0.1198
	OTU_13	Actinobacteria	Corynebacteriaceae_unass.	0.0422	0.0507

OTU_33	Bacilli	Staphylococcus	0.0195	0.0277
OTU_36	Bacilli	Lactobacillus	0.0128	0.0287
OTU_7	Fusobacteriia	Leptotrichia	0.0121	0.0058
OTU_62	Bacilli	Lactobacillus	0.0041	0.0121
OTU_1	Bacilli	Streptococcus	0.0072	0.0066
OTU_18	Bacteroidia	Bergeyella	0.0048	0.0027
OTU_5	Bacteroidia	Porphyromonas	5.00E-04	0.0018
OTU_88	Gammaproteobacteria	Conchiformibius	0	
OTU_235	Gammaproteobacteria	Pasteurellaceae_unass.	0	
OTU_200	Gammaproteobacteria	Pasteurellaceae_unass.	0	
OTU_146	Gammaproteobacteria	Rodentibacter	0	
OTU_4	Gammaproteobacteria	Rodentibacter	0.0035	
OTU_42	Gammaproteobacteria	Rodentibacter	0	
OTU_81	Gammaproteobacteria	Rodentibacter	0	
OTU_14	Gammaproteobacteria	Rodentibacter	0	
OTU_3	Gammaproteobacteria	Rodentibacter	0.0016	
OTU_70	Gammaproteobacteria	Rodentibacter	0	
OTU_60	Gammaproteobacteria	Rodentibacter	0	
OTU_1022	Gammaproteobacteria	Rodentibacter	0	
OTU_21	Gammaproteobacteria	Rodentibacter	0.1158	
OTU_122	Gammaproteobacteria	Rodentibacter	0	
OTU_670	Gammaproteobacteria	Rodentibacter	0	
OTU_8	Gammaproteobacteria	Rodentibacter	0.0098	
OTU_116	Gammaproteobacteria	Rodentibacter	0	
OTU_32	Gammaproteobacteria	Pasteurellaceae_unass.	0	
OTU_9	Gammaproteobacteria	Pasteurellaceae_unass.	1.00E-04	
OTU_74	Gammaproteobacteria	Rodentibacter	0	
OTU_378	Gammaproteobacteria	Pasteurellaceae_unass.	0	
OTU_107	Gammaproteobacteria	Cardiobacteriaceae_unass.	0	
OTU_29	Bacteroidia	Porphyromonas	0	
OTU_23	Fusobacteriia	Fusobacterium	2.00E-04	
OTU_91	Fusobacteriia	Leptotrichia	0	
OTU_95	Fusobacteriia	Leptotrichia	0	
OTU_37	Fusobacteriia	Leptotrichiaceae_unass.	0.0048	
OTU_90	Negativicutes	Veillonella	0	
OTU_78	Bacilli	Lactobacillus	0.0061	
OTU_52	Bacilli	Lactobacillus	0.0056	
OTU_162	Bacilli	Streptococcus	0	
OTU_72	Bacilli	Streptococcus	1.00E-04	
OTU_6270	Bacilli	Streptococcus	0	
OTU_749	Bacilli	Streptococcus	0	
OTU_38	Bacilli	Streptococcus	0	
OTU_22	Bacilli	Streptococcus	0.0018	
OTU_43	Bacilli	Carnobacteriaceae_unass.	0.0139	
OTU_25	Bacilli	Carnobacteriaceae_unass.	0.0091	
OTU_16	Bacilli	Gemella	0.0019	
OTU_28	Bacilli	Gemella	0.0238	

OTU_115	Bacilli	Gemella	0	
OTU_217	Bacilli	Gemella	0	
OTU_58	Bacilli	Staphylococcus	0.0374	
OTU_15	Actinobacteria	Corynebacterium	0	
OTU_12	Actinobacteria	Corynebacterium_1	0	
OTU_19	Actinobacteria	Actinobacteria_unass.	0.0649	
OTU_448	Actinobacteria	Bifidobacteriaceae_unass.	0.0011	
OTU_203	Actinobacteria	Actinobacteria_unass.	2.00E-04	
OTU_50	Actinobacteria	Actinomyces	1.00E-04	
OTU_113	Alphaproteobacteria	Sphingomonas		0.0039
OTU_202	Alphaproteobacteria	Caulobacteraceae_unass.		6.00E-04
OTU_187	Gammaproteobacteria	Moraxellaceae_unass.		0.0014
OTU_173	Gammaproteobacteria	Burkholderia-Caballeronia-Paraburkholderia		0.0012
OTU_154	Gammaproteobacteria	Acidibacter		0.0018
OTU_44	Campylobacteria	Campylobacter		0.0295
OTU_159	Bacilli	Lactobacillus		0.0036
OTU_253	Sericytochromatia	Sericytochromatia_unass.		6.00E-04
OTU_155	Acidimicrobiia	Acidimicrobiia_unass.		0.002
OTU_466	Actinobacteria	Geodermatophilus		3.00E-04
OTU_49	Actinobacteria	Rhodococcus		3.00E-04
